# Reliability and validity of the Turkish version of the Vocal Fatigue Index1This study was given as an oral presentation at the 41th Turkish National Otorhinolaryngology and Head Neck Surgery Congress,Antalya, 2019.

**DOI:** 10.3906/sag-1908-105

**Published:** 2020-06-23

**Authors:** Seher ŞİRİN, Mehmet Fatih ÖĞÜT, Cem BİLGEN

**Affiliations:** 1 Department of Otorhinolaryngology-Head and Neck Surgery, Faculty of Medicine, Kocaeli University, Kocaeli Turkey; 2 Division of Audiology and Speech Disorders, Health Sciences Institute, Ege University, İzmir Turkey; 3 Department of Otorhinolaryngology-Head and Neck Surgery, Faculty of Medicine, Ege University, İzmir Turkey

**Keywords:** Voice, vocal fatigue, reliability, validity, quality of life

## Abstract

**Background/aim:**

The aim of this study is to evaluate the reliability and validity of the Turkish version of the Vocal Fatigue Index (VFI).

**Materials and methods:**

The study was conducted with 535 participants including 285 patients with voice disorders and 250 healthy controls. Internal consistency and test-retest reliability were calculated for the reliability analysis. The mean VFI factor scores of both groups were compared. The strength and direction of the relation between VFI and Voice-Related Quality of Life (V-RQOL) measure was evaluated for the validity analysis.

**Results:**

The Cronbach’s alpha coefficient of VFI factor scores was found to be 0.920 for tiredness and avoidance of voice use, 0.879 for physical discomfort with voice use, and 0.882 for improvement of symptoms with voice rest. The test-retest reliability revealed 0.877 for the tiredness and avoidance of voice use, 0.913 for the physical discomfort with voice use, and 0.820 for the improvement of symptoms with voice rest. When compared with healthy individuals, VFI factor scores were statistically significant higher in patients with voice disorders. The V-RQOL scores decreased significantly as the VFI scores increased.

**Conclusion:**

The Turkish version of the VFI is a good reliable and valid instrument for evaluating vocal fatigue symptoms in the Turkish-speaking community.

## 1. Introduction

Vocal fatigue is a commonly reported condition in voice disorders although there is no commonly accepted definition of the term [1,2]. Vocal fatigue may occur as a result of the use of a dysphonic voice during normal daily activity and further restricts communication activities. Vocal hyperfunction due to negative vocal behavior such as abuse and/or misuse of voice might also lead to vocal fatigue and chronic vocal hyperfunction may lead to vocal pathologies [2,3]. In addition, prolonged use of the voice may also be the cause of isolated vocal fatigue even if there is no underlying voice disorder and/or negative behavior [4–6]. Therefore, vocal fatigue is a complex and multifaceted concept which can occur as a cause, consequence, or associated condition in voice disorders and the pathogenic relationship between vocal fatigue and voice disorders is not clear. Due to the different underlying physiological and biomechanical mechanisms, the published definitions of vocal fatigue are variable. While Welham and Maclagan [7] described vocal fatigue as a precursor to vocal pathologies that developed as a result of increased vocal load or prolonged use of voice, Solomon [2] proposed to include the concept of rest and described this situation as a perception of an increased vocal effort that increases over time with voice use and improves with voice rest. 

In addition to the different underlying causative mechanisms, vocal fatigue may present itself with a wide variety of symptoms, such as decrease in voice quality, increased vocal effort, reduced pitch range, fatigue and tightness in the throat, physical tiredness following prolonged use of voice and improvement of symptoms with voice rest [2,7,8]. Therefore, the variety of the underlying mechanisms and reported symptoms leads to a lack of a universally accepted definition for vocal fatigue. Typically, voice patients use the term ‘hoarseness’ to describe their chief complaint, but hoarseness may have different meanings, including vocal fatigue, to the lay patients [9]. It is necessary to clarify what exactly ‘hoarseness’ means for patients and this may require direction by the physician [9]. Vocal fatigue symptoms are related to an individual’s perception of symptoms. Therefore, they cannot be measured by the physician using objective assessment methods. However, patient-reported outcome measures would be ideal for making this subjective assessment. A reliable and valid scale would make it possible for both patients and physicians to understand an individual’s vocal fatigue in terms of identifying, quantifying, and measuring the voice-related concern and its impact on the patients’ daily life [10]. 

Due to the lack of published research in this field, Nanjundeswaran et al. (2015) developed the Vocal Fatigue Index (VFI), which is a reliable and valid self-assessment tool containing 19 items based on self-reported symptoms that is able to identify and distinguish vocal fatigue symptoms in research and clinical practice [11]. The ultimate goal for research into vocal fatigue is to identify and understand the mechanisms leading to vocal fatigue and thus to develop rational treatments for it. The VFI evaluated vocal fatigue across three dimensions: tiredness and avoidance of voice use (first 11 items, factor 1), physical discomfort with voice use (next 5 items, factor 2), and improvement of symptoms with voice rest (last 3 items, factor 3). Each dimension has been developed to define a different concept under the main heading vocal fatigue. Therefore, interpretation of the VFI is based on these factor scores rather than total scores. It is a 5-point Likert-type scale and is scored from 0 to 4 (0 = never, 1 = rarely, 2 = sometimes, 3 = often, 4 = always). 

In order to compare scientific data obtained from different societies, it would be necessary to study and determine whether the measurement tools are reliable and valid for the target society with standardized procedures [12]. Cross-cultural adaptation, reliability, and validity studies of the VFI have been conducted in different languages [13,14]. There is no such scale in the Turkish language to measure the symptoms of vocal fatigue. 

The aim of this study is to establish a Turkish version of the VFI and to perform its reliability and validity analysis.

## 2. Materials and methods

### 2.1. Study design

For testing the reliability and validity of the Turkish version of VFI, a clinical, prospective study with a control group was designed after obtaining permission from the original author. The study was conducted following approval obtained from the Institutional Review Board of the University of Kocaeli Medical School (KU/GOKAEK, 2017 / 825-167). All individuals included in the study were informed about the content and purpose of the study and their written permission was obtained. All procedures performed in studies involving human participants were in accordance with the ethical standards of the institutional and/or national research committee and with the 1964 Helsinki declaration and its later amendments or comparable ethical standards.

### 2.2. Translation procedure 

The method proposed by Guillemin et al. was used in the translation procedure [15]. The original VFI scale was translated into Turkish by two translators who were informed about the importance of this study. One of the translators was bilingual. The translated scales were evaluated by two voice experts with knowledge and experience in this field, and were converted to a merged scale. The merged scale was then back-translated into English by an independent bilingual (Turkish to English) translator who was not involved in the English-to-Turkish translations. Following back-translation, sentences compliant with the original scale were accepted and those that were not compliant with the original version were processed again until harmony with the original version was achieved. A pilot study of the final Turkish version of the VFI was performed using 25 volunteers through face-to-face interviews. The Turkish language VFI was assessed for comprehensibility, readability, and typographical accuracy. After final correction, the scale was applied to the study participants (Appendix).

### 2.3. Selection of study participants

The evaluations of the individuals included in the study were performed in the otorhinolaryngology department. Participants were Turkish literate and over 18 years old and presented with a voice complaint. Their relatives and volunteers who did not have a voice complaint were included in the study as a control group. During the application of the scale, patients presenting with voice complaints were diagnosed through detailed laryngeal examination. Patients with a voice disorder that required immediate intervention were excluded. In addition, professional voice users (e.g., elite professionals (singers, artists), teachers, and call center employees) that may be prone to vocal fatigue due to prolonged use of voice were excluded from the study to prevent bias. 

### 2.4. Reliability analysis

For reliability analysis, it is recommended to take at least ten samples per item, although there is not a commonly accepted absolute definition of required sample size [16,17]. It was decided to include 15 participants per item because voice complaint is a common reason for admission in otolaryngology clinical practice and the diagnosis of voice disorders show great diversity. The study involved 535 participants, of whom 285 (53.3%) were patients with voice complaints and 250 (46.7%) were without voice complaints. 

A test-retest analysis was performed to determine the consistency of the scale and all participants were asked to complete the scale again within 5–15 days. Care was taken to keep the time interval long enough so that participants could not remember their initial answers and short enough to ensure that their complaint would not change. During the period between first and second completion of the VFI, the patients did not receive any treatment. 

### 2.5. Validity analysis

Participants with and without voice complaint were compared and the ability of the Turkish version of the VFI to identify vocal fatigue in patients with voice disorders was evaluated. Participants were asked to complete the voice-related quality of life (V-RQOL) scale, which has been adapted into Turkish and the reliability and validity has been confirmed and previously published [18]. The correlation between the Turkish version of the VFI and the V-RQOL scale were evaluated in order to test the construct validity.

### 2.6. Voice-related quality of life

V-RQOL is a self-administered measurement consisting of 10 items evaluating the impact of voice-related problems experienced during daily life [19]. It is a five-point Likert-type scale ranging from 1 (none, not a problem) to 5 (as bad as it can be). It evaluates two domains, with four items on social-emotional and six items on physical functioning subscales. In addition, the overall quality of life effect can also be calculated. Both domain and total V-RQOL scores were standardized to a scale of 0 to 100, with a higher number indicating a better voice-related QOL. Validity and reliability testing of V-RQOL in a Turkish population was performed by Tezcaner and Aksoy [18].

### 2.7. Statistical analysis

SPSS for Windows v22 (IBM Corp., Armonk, NY, USA) and MedCalc for Windows v19.2.0 (MedCalc Software, Ostend, Belgium) were used to analyze data. The number and percentage of participants in the categorical variables were expressed as (n), and (%), respectively, and as mean ± standard deviation (mean ± SD) for the numerical variables. In reliability analysis, the Cronbach alpha internal consistency coefficient was used to evaluate internal consistency and test-retest (the Pearson product-moment correlation) reliability coefficient was used to evaluate the stability of the test. In addition to the internal consistency of the VFI subscale totals, when any given item was deleted the corrected item/total correlation coefficients and the alpha coefficient were calculated using the Cronbach alpha internal consistency coefficient in order to evaluate the strength of each item. A Cronbach’s alpha coefficient greater than 0.70 was considered statistically significant, acceptable, and reliable. Corrected item/total correlation coefficient greater than 0.50 was considered significant. A test-retest reliability coefficient above 0.9 was considered excellent reliability and above 0.8 as good reliability. The construct validity of the VFI was examined by calculating Pearson’s correlation coefficient and determining the strength and direction of the relationship between V-RQOL and the VFI. Student’s t-test was used for independent samples. The receiver operating characteristic (ROC) curve analysis was used to determine scores for the area under the curve (AUC) as well as sensitivity and specificity for the assessment of diagnostic accuracy. An AUC of >0.90 was considered excellent discrimination, >80 as good, and >0.70 as fair. Statistical significance was accepted as P < 0.05.

## 3. Results

A total of 535 individuals, including 285 with voice complaints and 250 without voice complaints, were included in the study. The mean (±SD) age of all participants was 42.04 ± 14.63 (range: 18–81) years and 310 of them were female (57.9%). The mean (±SD) age of the group with voice complaints was 42.82 ± 15.16 years and the mean (±SD) age of the vocal healthy group was 41.16 ± 13.98 years (P = 0.304). One-hundred seventy four of the individuals with voice complaints were female (61.1%) compared to the 136 female individuals in the vocal healthy group (54.4%) (P = 0.120). The primary diagnoses of participants with voice complaints are shown in Table 1. 

**Table 1:** Patient diagnoses.

	n (%)
Vocal fold nodule	78 (27.4)
Vocal fold paralysis	45 (15.8)
Functional dysphonia	33 (11.6)
Vocal fold polyp	29 (10.2)
Reinke edema/edema	27 (9.5)
Presbyphonia	21 (7.3)
Vocal fold cyst	16 (5.6)
Larynx premalignant lesion	13 (4.6)
Sulcus vocalis	9 (3.1)
Others	14 (4.9)
Total	285 (100)

### 3.1. Reliability analysis

#### 3.1.1. Internal consistency

The strength of each and every item was determined with the corrected item/total correlation coefficient and it was observed that no item was below 0.50 (Table 2). The internal consistency coefficients (Cronbach’s alpha) of the remaining items when each item of the scale was deleted were above 0.791 (Table 2). The internal consistency coefficients of VFI factors showed an excellent internal consistency for tiredness and avoidance (Cronbach’s alpha = 0.920) and a high internal consistency for physical discomfort (Cronbach’s alpha = 0.879) and improvement of symptoms (Cronbach’s alpha = 0.882) (Table 3). 

**Table 2:** Mean and SD scores of each item, item/total correlations, and alpha coefficient (when item is deleted) in VFI.

Item	Mean	SD	Corrected item/total correlation	Alpha coefficient(when this item is deleted)
1	2.53	1.11	0.760	0.909
2	3.01	1.01	0.742	0.911
3	2.90	0.99	0.695	0.913
4	2.92	1.05	0.698	0.913
5	2.64	1.30	0.754	0.909
6	2.55	1.22	0.764	0.909
7	2.16	1.35	0.571	0.919
8	2.05	1.37	0.676	0,914
9	2.72	1.10	0.736	0.911
10	2.47	1.30	0.637	0.916
11	2.56	1.20	0.575	0.918
12	1.77	1.80	0.518	0.911
13	2.03	1.39	0.777	0.838
14	1.98	1.39	0.766	0.841
15	1.89	1.39	0.843	0.823
16	2.02	1.42	0.727	0.849
17	2.45	1.15	0.741	0.859
18	2.39	1.19	0.757	0.845
19	2.25	1.21	0.817	0.791

VFI: Vocal Fatigue Index

**Table 3:** Reliability analysis: internal consistency (Cronbach’s Alpha) and test-retest (Pearson’s r) reliability of VFI factor scores.

	Internal consistency	Test-retest reliability	
	Cronbach alpha	r	P
Tiredness and avoidance	0.920	0.877	<0.001
Physical discomfort	0.879	0.913	<0.001
Improvement of symptoms	0.882	0.820	<0.001

The correlation coefficients between each factor of VFI were examined. Tiredness and avoidance factor and physical discomfort factor scores were strongly correlated with each other (r = 0.621 and P < 0.001). The improvement of symptoms with rest factor scores poorly correlated with tiredness and avoidance factor scores (r = 0.291 and P < 0.001) and physical discomfort factor scores (r = 0.372 and P < 0.001).

#### 3.1.2. Test-retest reliability

The mean time interval between the completion of the two scales was 7 days (SD ± 2.8). Strong test-retest reliability was seen for all three factors: tiredness and avoidance (r = 0.877), physical discomfort (r = 0.913), and improvement of symptoms with rest (r = 0.820) (Table 3).

### 3.2. Validity analysis 

When compared with participants without voice complaint, the mean VFI factor scores were statistically significantly higher in patients with voice disorder (P < 0.001). While the difference in mean scores for improvement of symptoms with rest factor was approximately two-fold higher, the difference in the mean tiredness and avoidance factor and physical discomfort factor scores were approximately five-fold higher. The mean (±SD) scores of participants with and without voice complaint for each factor are shown in Table 4. 

**Table 4:** Mean and SD of VFI Factor scores for patients with voice complaint and participants without voice complaint.

	Patients with voice complaint	Participants without voice complaint	P-value
VFI factors	Mean ± SD	Mean ± SD	
Tiredness and avoidance	28.56 ± 9.79	5.46 ± 5.66	<0.001
Physical discomfort	9.71 ± 6.12	1.97 ± 2.89	<0.001
Improvement of symptoms	7.10 ± 3.21	3.54 ± 3.95	<0.001

The strength and direction of the relation between the VFI factor scores and the V-RQOL domains and overall scores were calculated in order to examine the construct validity of the Turkish version of the VFI. A significant negative correlation between the three factors of the VFI and the V-RQOL scale was observed. As the VFI factor scores increased, the overall V-RQOL scores decreased with a very strong correlation with tiredness and avoidance factor scores (r = –0.809, P < 0.001), a moderate correlation with physical discomfort factor scores (r = –0.512, P < 0.001), and a very poor correlation with improvement of symptoms with voice rest factor scores (r = –0.147, P = 0.014). The calculated correlation coefficients are shown in Table 5. 

**Table 5:** Pearson correlation analysis of VFI and V-RQOL in patients with voice complaint.

	V-RQOLPhysical functioning		V-RQOLSocio-emotional		V-RQOLTotal	
VFI factors	r	P	r	P	r	P
Tiredness and avoidance	–0.803	<0.001**	–0.700	<0.001**	–0.809	<0.001**
Physical discomfort	–0.518	<0.001**	–0.427	<0.001**	–0.512	<0.001**
Improvement of symptoms	–0.201	0.001**	–0.052	0.388	–0.147	0.014*

VFI: Vocal Fatigue Index; V-RQOL: Voice-related quality of life

ROC curve analysis was performed to assess the diagnostic accuracy of the VFI. The AUC of each factor were as follows: 0.962 (95% confidence interval [CI]: 0.942–0.976, P < 0.001) for factor 1, 0.862 (95% CI: 0.829–0.890, P < 0.001) for factor 2 and 0.761 (95% CI: 0.723–0.797, P < 0.001) for factor 3 (Figure). The sensitivity and specificity of each factor with the cut-off values were as follows; 90.1% and 94.4% for factor 1 scores > 16, 76.1% and 87.6% for factor 2 scores > 4, and 78.9% and 69.6% for factor 3 scores < 4. 

**Figure:**
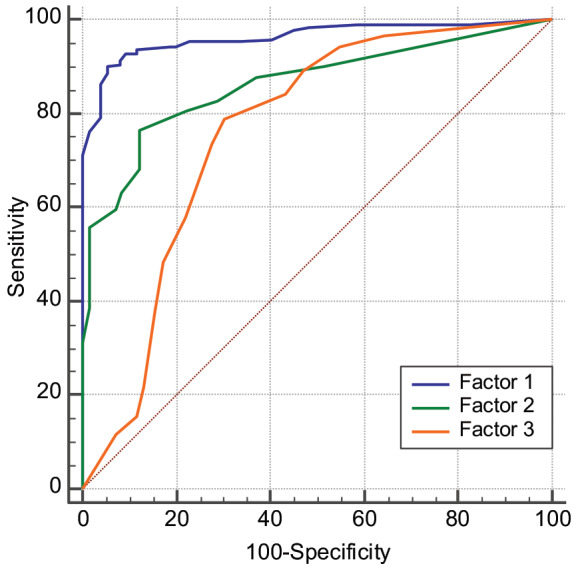
ROC curve analysis for the three factor scores of Turkish VFI to discriminate individuals with and without voice fatigue. ROC: Receiver operating characteristics; VFI: Vocal Fatigue Index

## 3. Discussion

Although the term ‘vocal fatigue’ is frequently used in both clinical practice and the literature, the lack of a universally accepted definition and standardized assessment method leads to challenges in its identification and therefore management. To the best of our knowledge, the VFI is the first standardized assessment tool to address vocal fatigue in detail based on self-reported symptoms. Patient-reported outcome measures provide valuable information for both patients and physicians in terms of identifying, measuring, and monitoring voice-related concerns and their impact on daily life [10]. Standardized measurements are also important for comparing data from different sociolinguistic cultures and establishing a global standard. Prior to the use of any scale designed to elucidate patient-reported outcome measures in research and clinical practice, the usability should be tested using accepted procedures in the target language and society. Reliability and validity are key features for standardization of a scale [12]. 

The reliability tests of the VFI developed by Nanjundeswaran et al. included a total of 270 individuals, of whom 200 had voice disorder. It was reported that the internal consistency coefficients were 0.93 for the fatigue and avoidance factor, 0.89 for physical discomfort factor, and 0.82 for improvement of symptoms with rest factor [11]. Naderifar et al. reported internal consistency coefficients of 0.95, 0.86, and 0.83, respectively, according to their study of the VFI after it was translated and adapted into Persian [13]. Athira and Devadas reported these coefficients to be 0.922, 0.923, and 0.925, respectively, in their VFI, adapted into the Malayalam language, and tested in teachers with voice disorders [14]. In our study, the reliability of each and every item in the Turkish version of the VFI was evaluated by calculating Cronbach’s alpha internal consistency coefficient. The internal consistency coefficients of the VFI were 0.920 for tiredness and avoidance factor, 0.879 for physical discomfort factor, and 0.882 for improvement of symptoms with rest factor. In addition, when the reliability of each item was evaluated, no item was deleted during the cross-cultural adaptation. In our study, test-retest reliability was 0.877 for the tiredness and avoidance factor, 0.913 for the physical discomfort factor, and 0.820 for the improvement of symptoms with rest factor. According to these findings, it can be concluded that the Turkish version of VFI has good reliability.

The primary aim of Nanjundeswaran et al. was to be able to reliably identify vocal fatigue symptoms in individuals with this condition while developing the VFI [11]. Therefore, they evaluated the ability of the VFI to differentiate patients with voice complaints from control subjects without voice complaints and reported that the scale was able to detect symptoms of vocal fatigue in individuals with voice disorders [11]. Similarly, in the Malayalam and Persian version studies, significantly higher factor scores were reported in patients with voice disorders compared to individuals without voice complaint [13,14]. Our study results were consistent with those of previous studies, and higher significant factor scores were obtained in patients with voice disorders. It can be concluded that the Turkish version of VFI is able to identify vocal fatigue symptoms in patients with voice disorders. 

The VFI is not developed as a unitary construct instrument [11]. The factors forming the VFI were determined as different concepts in order to provide a more comprehensive assessment and capture different aspects of voice fatigue. Therefore, its interpretation is based on factor scores rather than total scores. Additionally, the questions in factors 1 and 2 are worded negatively whereas the three items included in factor 3 are worded positively. While an increase in factor 1 and 2 scores indicates an increase in vocal fatigue symptoms and/or severity and the decrease in factor 1 and 2 scores indicates fewer vocal fatigue symptoms and/or severity; the increase in factor 3 scores does not mean that vocal fatigue symptoms are less. A decrease in factor 3 scores indicates fatigue which does not improve with voice rest and an increase in factor 3 scores indicates vocal fatigue symptoms which improve with voice rest. Besides, the correlation coefficients between three factors of VFI were reported as quite different in the original version, indicating that they do not contribute to the same degree in VFI (factors 1,3 = 0.39; factors 2,3 = 0.34; and factors 1,2 = 0.59) [11]. When the difference in the mean factor scores in patients with voice disorders were evaluated, the increase rate in factor 3 scores was not as high as that in factor 1 and 2 scores based on the mean scores of the participants without voice complaint which was almost five-fold higher for factors 1 and 2 but only 1.3 times higher for factor 3 [11]. Nanjundeswaran et al. reported that the same consistency in symptoms scores could not be observed in all individuals and mixed results might be because of different mechanisms of vocal fatigue in each individual [11]. The results of our study were consistent with the original report. The improvement of symptoms with voice rest factor scores were approximately two times higher and the mean scores of the other two factor scores were approximately five times higher in patients with voice disorders compared to subjects without voice disorder. When the relationship between the scores of each factor of the Turkish version of the VFI was evaluated, although factor 1 was strongly correlated with factor 2 scores; the factor 3 scores showed a moderate correlation with factor 1 and a weak correlation with factor 2 scores (factors 1,2 = 0.621; factors 1,3 = 0.372; factors 2,3 = 0.291 and P < 0.001 for all). According to these results, it would be more appropriate to evaluate vocal fatigue aspects based on the separate three factor scores instead of calculating the total score when using the Turkish version of VFI.

One of the methods used to evaluate the validity of a measure is to examine its correlation with external tests. As no vocal fatigue instrument had been developed before and the Turkish V-RQOL is the closest validated test in this field, it was decided to use this measure as a comparison test. The V-RQOL measure was developed to evaluate the impact of voice-related concern on an individual’s daily life and was not specifically designed for any specific category of voice disorders and/or voice complaint. The VFI provides information on the degree of limitation in daily activity due to vocal fatigue by means of the tiredness and avoidance factor. Thus, an inverse correlation with quality of life was expected. Correlation coefficients between VFI factor scores and the V-RQOL scores were calculated in order to evaluate the construct validity of the VFI. All three VFI factor scores showed a significant inverse correlation with the overall V-RQOL scores and factor 1 showed the strongest correlation, as expected (r = –0.809 and P < 0.001 for tiredness and avoidance). As the symptoms and severity of vocal fatigue increased and the VFI factor scores increased, the overall V-RQOL scores significantly decreased. The variation among the factor score correlation coefficients, especially the weak correlation for factor 3 scores (r = –0.147; P = 0.014 for improvement of symptoms with rest) can be explained by the VFI not being a unitary construct instrument. In such a case, it would be expected that in the correlation of VFI factors with the external test, results consistent with the individual factor correlations would be found. A similar difference was observed in the correlation coefficients between the three factors of the VFI. Therefore, although factor 3 seems to be poorly correlated with the V-RQOL scores, our construct validity analysis results for all the three factors were consistent with the VFI internal dynamics and were as expected. Based on these findings, it can be concluded that the Turkish version of VFI is a valid scale in patients with voice disorder.

The discriminative ability of the VFI was reported as excellent by Nanjundeswaran et al. (AUC: 0.91, 90% sensitivity, and 90% specificity for each factor; cut-of values for factor 1, 2, and 3 were ≥24, ≥7, and ≤7, respectively). The German version was reported with fairly good discriminative scores ( factor 1: AUC; 0.851, 76.2% sensitivity, 90.0% specificity with a cut-of value of ≥15.5; factor 2: AUC; 0.769, 71.5% sensitivity, 81% specificity with a cut-of value of ≥2.5; factor 3: AUC; 0.674, 50.5% sensitivity, 80% specificity with a cut-of value of ≤7.5) [20]. In our study, the discriminative scores were excellent for factor 1, good for factor 2, and fair for factor 3 (factor 1: AUC; 0.962, 90.1% sensitivity, 94.4% specificity with a cut-of value of >16; factor 2: AUC; 0.862, 76.1% sensitivity, 87.6% specificity with a cut-of value of >4; factor 3: AUC; 0.761, 78.9% sensitivity, 69.9% specificity with a cut-of value of <4). The cut-of values of our study were lower than the original version and closer to the German version, with better sensitivity and specificity, suggesting that VFI scores exceeding these thresholds indicate vocal fatigue with optimum sensitivity and specificity. According to these findings, it can be concluded that the Turkish version of the VFI has a very good accuracy and discriminative ability in subjects with and without vocal fatigue. 

The Turkish version of VFI was tested in patients with voice disorders and compared with healthy controls with no vocal fatigue precipitating factors. Future studies with the Turkish version of the VFI that address its ability to identify and quantify vocal fatigue symptoms in individuals with high vocal demands such as elite professionals, teachers, call center employees and Islamic religious officials, who are susceptible to vocal fatigue even without vocal pathology, might contribute to a better understanding of this very prevalent condition and therefore to its management.

In conclusion, the Turkish version of VFI is a reliable and valid measurement tool that can be used for identifying, quantifying, and evaluating vocal fatigue symptoms in the Turkish-speaking community.

## Acknowledgments

The authors would like to thank Mehmet Akif Kılıç for his valuable expert opinion during the translation procedure, Firdevs Alioğlu for her statistical support, and Jeremy Jones for his linguistic revision. No financial support was received for this paper.

## Conflict of interest

The authors declare that they have no conflict of interest.

## Appendix

**Table A1:** SES YORGUNLUĞU ÖLÇEGİ (Vocal Fatigue Index)

Bölüm 1						
1	Sesimi bir süre kullandıktan sonra daha fazla konuşmak istemiyorum.	0	1	2	3	4
2	Çok konuştuğumda sesimin yorulduğunu hissediyorum.	0	1	2	3	4
3	Konuşurken sürekli artan bir şekilde çaba sarf ediyorum.	0	1	2	3	4
4	Kullandıkça sesim kısılıyor.	0	1	2	3	4
5	Konuşmak çaba gerektiren bir işmiş gibi geliyor.	0	1	2	3	4
6	Bir süre konuştuktan sonra genellikle konuşmamı sınırlama ihtiyacı duyuyorum.	0	1	2	3	4
7	Fazla konuşma gerektiren sosyal ortamlardan uzak duruyorum.	0	1	2	3	4
8	Bir iş günü sonrasında ailemle konuşamayacakmışım gibi geliyor.	0	1	2	3	4
9	Bir süre konuştuktan sonra konuşmak için daha fazla çaba sarf ediyorum.	0	1	2	3	4
10	Konuşurken sesimi duyurmakta zorlanıyorum.	0	1	2	3	4
11	Bir süre konuştuktan sonra sesim cılızlaşıyor.	0	1	2	3	4
Bölüm 2						
1	Sesimi çok kullandığım günün sonunda boynumun ağrıdığını hissediyorum.	0	1	2	3	4
2	Sesimi çok kullandığım günün sonunda boğazımda ağrı hissediyorum.	0	1	2	3	4
3	Çok konuştuğumda ses tellerimde acı hissediyorum.	0	1	2	3	4
4	Sesimi kullandığımda boğazım sızlıyor.	0	1	2	3	4
5	Sesimi kullandığımda boynumda rahatsızlık duyuyorum.	0	1	2	3	4
Bölüm 3						
1	Dinlendikten sonra sesim daha iyi oluyor.	0	1	2	3	4
2	Ses çıkarmak için gösterdiğim çaba dinlendikçe azalıyor.	0	1	2	3	4
3	Ses kısıklığım dinlendikçe düzeliyor.	0	1	2	3	4
